# *Staphylococcus*-Induced Bacteriospermia In Vitro: Consequences on the Bovine Spermatozoa Quality, Extracellular Calcium and Magnesium Content

**DOI:** 10.3390/ani11113309

**Published:** 2021-11-19

**Authors:** Michal Ďuračka, Kamila Husarčíková, Mikuláš Jančov, Lucia Galovičová, Miroslava Kačániová, Norbert Lukáč, Eva Tvrdá

**Affiliations:** 1 Institute of Applied Biology, Faculty of Biotechnology and Food Sciences, Slovak University of Agriculture in Nitra, Tr. A. Hlinku 2, 94976 Nitra, Slovakia; norbert.lukac@uniag.sk (N.L.); evina.tvrda@gmail.com (E.T.); 2 Department of Biotechnology, University of Chemistry and Technology Prague, Technická 5, 16628 Prague, Czech Republic; husarcikova.kamila@gmail.com; 3 Department of Animal Morphology, Physiology and Genetics, Faculty of Agrisciences, Mendel University in Brno, Zemědelská 1, 61300 Brno, Czech Republic; mikulasjancov@gmail.com; 4 Institute of Horticulture and Landscape Engineering, Slovak University of Agriculture in Nitra, Tr. A. Hlinku 2, 94976 Nitra, Slovakia; l.galovicova95@gmail.com (L.G.); kacaniova.miroslava@gmail.com (M.K.); 5 Department of Bioenergetics, Food Analysis and Microbiology, Institute of Food Technology and Nutrition, University of Rzeszow, Cwiklinskiej 1, 35-601 Rzeszow, Poland

**Keywords:** *Staphylococcus*, sperm quality, bull sperm, bacteria, bacterial contamination, oxidative stress, DNA fragmentation

## Abstract

**Simple Summary:**

Livestock semen is often contaminated by opportunistic bacterial pathogens originating from an intrinsic environment of the urogenital tract. Particularly, species classified in the Staphylococcus genus are predominantly represented in bovine ejaculates. Until recently, it was believed that these are a negligible part of the bovine ejaculate; however, recent studies revealed their potentially adverse effects on the sperm quality. Hereby, we simulated staphylococcal infection of bovine semen under laboratory conditions and analyzed its consequences on the sperm quality.

**Abstract:**

Bacterial contamination of bovine ejaculates intended for artificial insemination may be reflected in a significant economic loss due to unsuccessful fertilization as well as health issues of the recipients. The *Staphylococcus* genus represents a large part of bacteriocenosis of bovine ejaculates. Therefore, this study aims to get a closer look on the effects of *Staphylococcus*-induced bacteriospermia under in vitro conditions on bovine sperm quality. Prior to inducing bacteriospermia, spermatozoa were separated from each ejaculate using Percoll^®^ Plus gradient medium in order to limit the effects only to the selected bacterial species. Seven *Staphylococcus* species previously isolated from bovine semen were used for our experiments at a turbidity of 0.5 McFarland (equivalent to 1.5 × 10^8^ colony-forming units per mL). The contaminated semen samples were incubated at 37 °C and at times of 0, 2, and 4 h, motility, mitochondrial membrane potential, reactive oxygen species (ROS) generation, sperm DNA fragmentation, and magnesium (Mg) and calcium (Ca) extracellular concentration were analyzed and compared with the control group (uncontaminated). The results showed no significant changes at the initial measurement. However, significant adverse effects were observed after 2 h and 4 h of incubation. Most notably, the presence of *S. aureus*, *S. warneri*, *S. kloosii*, and *S. cohnii* caused a significantly increased ROS production, leading to sperm DNA fragmentation, changes in the mitochondrial membrane potential, and a decreased sperm motility. Furthermore, the presence of *Staphylococcus* species led to lower extracellular concentrations of Mg and Ca. In conclusion, the overgrowth of *Staphylococcus* bacteria in bovine semen may contribute to oxidative stress resulting in sperm DNA fragmentation, altered mitochondrial membrane potential, and diminished sperm motility.

## 1. Introduction

Opportunistic pathogens were previously debated as an integral part of bovine ejaculates of clinically healthy bulls. Recent studies on the presence of bacteria and their impact on the semen quality revealed that both the bacterial composition as well as their load may affect cellular structures involved in the fertilizing potential of bovine spermatozoa. Particularly, species representing the *Staphylococcus* genus seem to significantly contribute to a deteriorated sperm quality [1,2]. Species of the *Staphylococcus* genus serve as a reservoir of antimicrobial resistance genes representing a threat to animal as well as human health [3]. World Health Organization (WHO) defines *Staphylococcus aureus* (methicillin-resistant, vancomycin-intermediate, and resistant) as a high priority in the list for research and development of new antibiotics [4].

Bacteriospermia, defined as an increased concentration of bacteria in semen, is defined in human andrology as growth of >10^3^ pathogenic bacteria or >10^4^ non-pathogenic bacteria [5]. Although bacteriospermia is not exactly defined in animal andrology, mean values of colony-forming units (CFU) of bull semen are commonly reported to oscillate between 10^3^ and 10^6^ on average [2,6,7,8].

The pathophysiology behind bacteriospermia includes direct and indirect sperm damage mechanisms. Oxidative stress (OS) represents an important indirect factor associated with the harmful effects of bacteriospermia. So far, polymorphonuclear leukocytes are considered as the main source of reactive oxygen species (ROS) during bacteriospermia [9]. According to Fraczek et al. [10], a direct negative effect of bacteria lies in a mitochondrial regulated cell death. Both detrimental mechanisms are associated with sperm DNA fragmentation and, thereby, in the fertilization potential. Our research group revealed that not only the structure and function of spermatozoa may be affected by the bacterial load, but also the biochemical composition of seminal plasma is dependent on the amount of bacteria present in the specimen. Particularly, a strong significant correlation was recorded between CFU and magnesium concentration [11]. A previous study also reported that magnesium, calcium, as well as their natural ratio in seminal plasma are necessary for a proper function of sperm [12].

This study examines the effects of bacteriospermia induced by *Staphylococcus* species isolated from bovine semen under in vitro conditions. Sperm motility, plasma membrane integrity, mitochondrial membrane potential, ROS concentration, and DNA fragmentation were determined immediately following the initiation of the experiment, as well as after 2 and 4 h of bacteria/sperm co-incubation at 37 °C. Moreover, the extracellular content of magnesium and calcium were investigated.

## 2. Materials and Methods

### 2.1. Semen Collection and Gradient Separation

In this study, a total of 5 ejaculates were collected from 5 healthy Holstein Friesian breeding bulls (one ejaculate from each animal) and provided by the Slovak Biological Services (Nitra, Slovakia) during a regular collection schedule. Only samples which met the minimal criteria (70% spermatozoa motility) were used in this study. Immediately after transport (approximately 20 min.; held in thermos pre-warmed to 37 °C), semen was processed using the Percoll^®^ Plus (PP) density gradient medium (Sigma-Aldrich, St. Louis, MO, USA) and the sperm pellet was recovered and used for the experiment. A single-layer centrifugation with 70% gradient media was performed according to Martins Jr. et al. [13]. Approximately 1 × 10^9^ cells were gently layered on the top of PP (9 mL, 70%) which was previously diluted with Sp-TALP according to Parrish et al. [14]. Falcon tubes were centrifuged at 839× *g* for 13 min. The formed layers were individually discarded and the pellet was subsequently used for the experiments.

### 2.2. Study Design

Spermatozoa were diluted to 50 × 10^6^ cells/mL with Dulbecco’s Phosphate Buffered Saline with MgCl_2_ and CaCl_2_ (DPBS; Sigma-Aldrich, St. Louis, MO, USA) containing 0.5 McFarland units of *Staphylococcus* (equivalent to 1.5 × 10^8^ colony-forming units (CFU)/mL). Previous standardization experiments showed that this concentration is high enough to record negative effects to spermatozoa motility, but not too high to monitor which of the *Staphylococcus* species represent a high risk to the sperm quality. The isolates originated from previously gathered bovine semen samples. Seven experimental groups contained following isolates of the *Staphylococcus* genus: *S. aureus*, *S. haemolyticus*, *S. warneri*, *S. lentus*, *S. epidermidis*, *S. kloosii*, and *S. cohnii*. The control group did not contain any added bacteria. The analyses were performed after 2 and 4 h of co-incubation at 37 °C.

### 2.3. Sperm Motility Analysis

Computer-aided sperm analysis (CASA; Version 14.0 TOX IVOS II.; Hamilton-Thorne Biosciences, Beverly, MA, USA) was used in order to perform sperm motility analysis. Ten microliters of each sample were placed into the Makler counting chamber (depth 10 µm; Sefi Medical Instruments, Haifa, Israel) pre-warmed to 37 °C. The total sperm motility was objectively evaluated and expressed as the percentage of spermatozoa with a velocity of ≥5 µm/s. The system was preset as follows: frame rate—60 Hz; minimum contrast—20; static head size—0.25–5.00; static head intensity—0.40–2.00; static elongation—20–100; default cell size—4 pixels; and default cell intensity—40. At least, 30 cells were subjected to the motility analysis in a single field while 10 fields were evaluated during each motility measurement.

### 2.4. Plasma Membrane Integrity

The plasma membrane integrity was determined using the eosin-nigrosin staining assay. The diluted spermatozoa (5 µL) were stained with eosin (5%; 10 µL) and subsequently nigrosine (10%; 10 µL). Smears were left to dry at room temperature. At least 200 cells were assessed under a bright-field microscope (×1000; Nikon ECLIPSE E100, Tokyo, Japan). White sperm heads were counted as cells with an intact membrane, while pink sperm heads were evaluated as cells with a damaged membrane. The results are expressed as the percentage of the cells with preserved membrane integrity [2].

### 2.5. Mitochondrial Membrane Potential Assay

The mitochondrial membrane potential (MMP) was determined using a fluorescence spectroscopy with the help of lipophilic cation dye (JC-1 Mitochondrial Membrane Potential Assay Kit, Cayman, Ann Arbor, MI, USA). High MMP was characterized by the formation of polymers and detected by the emission of orange/red fluorescence (excitation/emission: 535/595 nm). Low MMP was defined by the monomeric form of the dye and detected by green fluorescence. One million cells were stained with 5 µL of the working solution of dye (5 µM) for 20 min at 37 °C. Prior the analysis, each sample was centrifuged (400× *g*, 5 min, room temperature) and washed with Assay buffer (200 µL) twice. After a third centrifugation, the pellet was mixed with 100 µL of assay buffer, and the fluorescence was measured on a dark 96-well microplate using the Glomax Multi^+^. The results are defined as the ratio of polymers and monomers [15].

### 2.6. Quantification of Reactive Oxygen Species

Reactive oxygen species was measured by a 96-well microplate chemiluminescent assay using luminol as the probe. One hundred microliters of Dulbecco’s phosphate buffered saline (DPBS) was added to the blank, negative, and positive controls. The tested samples (100 µL) as well as negative and positive controls were treated with 5 mM of luminol (5-amino-2,3-dihydro-1,4-phthalazinedione). Hydrogen peroxide (30%; 9.8 M; Sigma-Aldrich, St. Louis, MO, USA) was added to the positive controls. The chemiluminescence was measured using the Glomax Multi+ (Promega, Madison, WI, USA), a combined spectro-fluoro-luminometer. The results are interpreted as the relative light units/s/1 × 10^6^ spermatozoa [16].

### 2.7. Chromatin-Dispersion Test

The Sperm-Halomax^®^ kit (Halotech DNA, Madrid, Spain) was used to determine sperm DNA damage. The dispersion of sperm chromatin of each sample (diluted to 15–20 × 10^6^ sperm/mL) was evaluated under a fluorescent microscope (Leica DMI6000B, Wetzlar, Germany). Firstly, spermatozoa were fixed on a glass slide in an agarose matrix, treated with a lysis solution (5 min), washed in distilled water (5 min), and dehydrated in a 2-min cycles of ethanol baths (70%, 90%, and 100%). The sperm cells were stained with 2 µg/mL of SYBR Green (Sigma-Aldrich, St. Louis, MO, USA) in Vectashield (Vector Laboratories, Burlingame, CA, USA). The big dispersion around the sperm head characterizes the DNA fragmentation. The small compact dispersion around the sperm head characterizes the intact DNA. The results are interpreted as the percentage of sperm cells with fragmented DNA [2].

### 2.8. Extracellular Concentration of Magnesium

Extracellular magnesium (Mg) concentration was analyzed photometrically using a diagnostic kit (Magnesium XL FS, DiaSys Diagnostic Systems, Holzheim, Germany). The principle of this test lies in the reaction between xylidyl blue (110 µmol/L) with Mg ions in alkaline solution which results in a purple complex. Ten microliters of blank/standard/tested sample were mixed with 1000 µL of reagent. After 5 min at room temperature, the absorbance was measured at 520–628 nm by RX Monza biochemical analyzer (Randox Laboratories, Crumlin, UK). The results are defined in mM/L.

### 2.9. Extracellular Concentration of Calcium

Extracellular calcium (Ca) concentration was analyzed photometrically using a diagnostic kit (Calcium AS FS, DiaSys Diagnostic Systems, Holzheim, Germany). The principle of this test lies in the reaction with arsenazo III (120 µmol/L) at neutral pH which results in a blue complex. Ten microliters of the blank/standard/tested sample were mixed with 1000 µL of reagent. After 5 min at room temperature, the absorbance was measured at 650 nm by an RX Monza biochemical analyzer (Randox Laboratories, Crumlin, UK). The results are defined in mM/L.

### 2.10. Statistical Analysis

Statistical analysis was performed by the GraphPad Prism program (version 8.0 for Mac, GraphPad Software Inc., San Diego, CA, USA). One-way ANOVA followed by the Dunnett’s multiple comparisons test was performed in order to compare each group with a negative control (without added bacteria). Results are stated as arithmetic means ± standard errors of means. The significance level for the comparative analysis was set at * *p* < 0.05, ** *p* < 0.01, and *** *p* < 0.001.

## 3. Results

### 3.1. Effect of Staphylococcus-Induced Bacteriospermia on Total Sperm Motility

**Figure 1:**
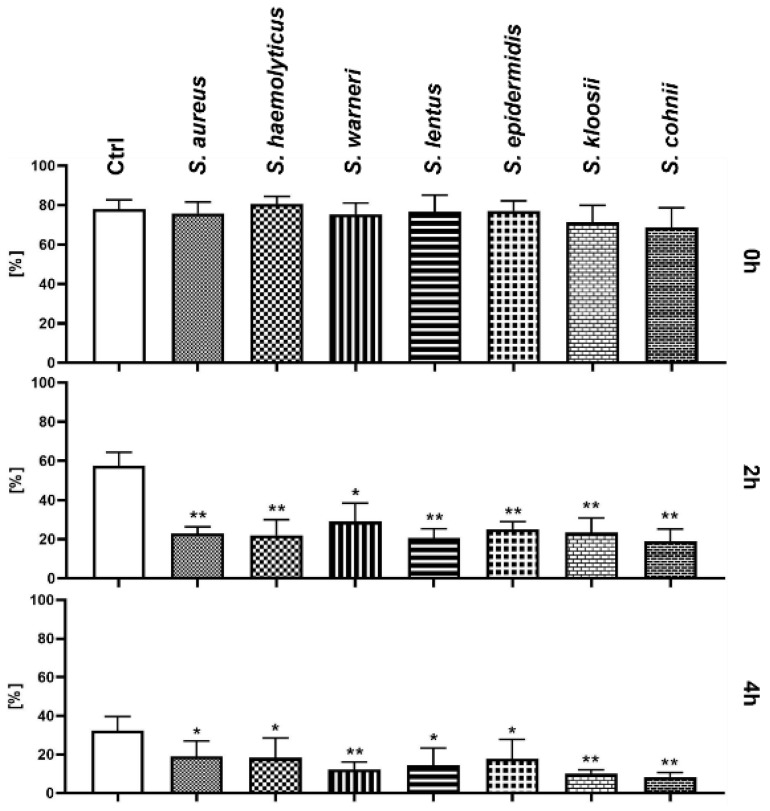
Changes in sperm motility during the 4 h bacteria/sperm co-incubation in vitro at 37 °C. Each group treated with *Staphylococcus* species was compared with the Ctrl group with no added bacteria. Each bar represents the mean percentage of motile sperm cells (≥5 µm/s) ± S.E.M. Five individual experiments were performed to obtain these data. The level of statistical significance was set at * *p* < 0.05; ** *p* < 0.01.

No significant changes were recorded at the beginning of these experiments when compared with the Ctrl group. After 2 h of the co-incubation, spermatozoa motility significantly decreased (*p* < 0.01) in each experimental group treated with *Staphylococcus* species (*p* < 0.05 in case of *S. warneri*). Nevertheless, there were significant differences in each experimental group after 4 h of the co-incubation (Figure 1); the most significant decrease in spermatozoa motility was observed following treatment with *S. warneri*, *S. kloosii*, and *S. cohnii* (*p* < 0.01).

### 3.2. Effect of Staphylococcus-Induced Bacteriospermia on Plasma Membrane Integrity

**Figure 2:**
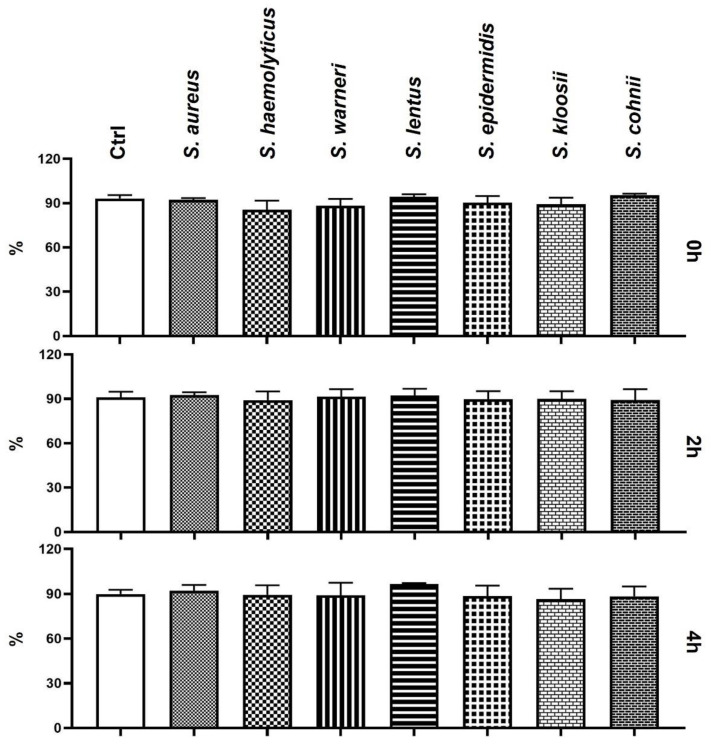
Changes in plasma membrane integrity during the 4-h bacteria/sperm co-incubation in vitro at 37 °C. Each group treated with *Staphylococcus* species was compared with the Ctrl group with no added bacteria. Each bar represents the mean percentage of sperm cells with intact plasma membrane ± S.E.M. Five individual experiments were performed to obtain these data.

The eosin–nigrosin staining assay did not reveal any differences in the sperm plasma membrane integrity between Ctrl and experimental groups throughout the duration of the experiment (Figure 2).

### 3.3. Effect of Staphylococcus-Induced Bacteriospermia on Sperm Mitochondrial Membrane Potential

**Figure 3:**
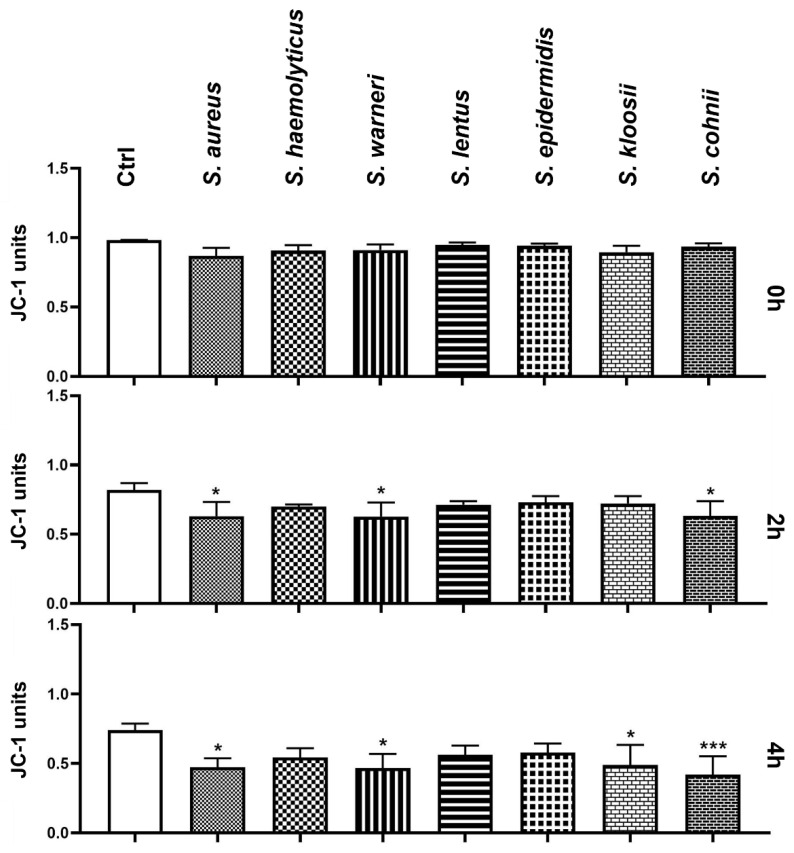
Changes in sperm mitochondrial membrane potential during the 4 h bacteria/sperm co-incubation in vitro at 37 °C. Each group treated with *Staphylococcus* species was compared with the Ctrl group with no added bacteria. Each bar represents the mean ratio of JC-polymers and JC-monomers ± S.E.M. Five individual experiments were performed to obtain these data. The level of statistical significance was set at * *p* < 0.05; *** *p* < 0.001.

The mitochondrial membrane potential was not affected by the presence of bacteria during the initial measurement (Figure 3). However, the 2 h measurement showed a significant decrease in the groups infected by *S. aureus*, *S. warneri*, and *S. cohnii* (*p* < 0.05) when compared to the Ctrl group. The last analysis revealed significant changes in the mitochondrial membrane in the groups with added *S. aureus*, *S. warneri*, *S. kloosii* (*p* < 0.05), and *S. cohnii* (*p* < 0.001).

### 3.4. Effect of Staphylococcus-Induced Bacteriospermia on Global Reactive Oxygen Species Production

**Figure 4:**
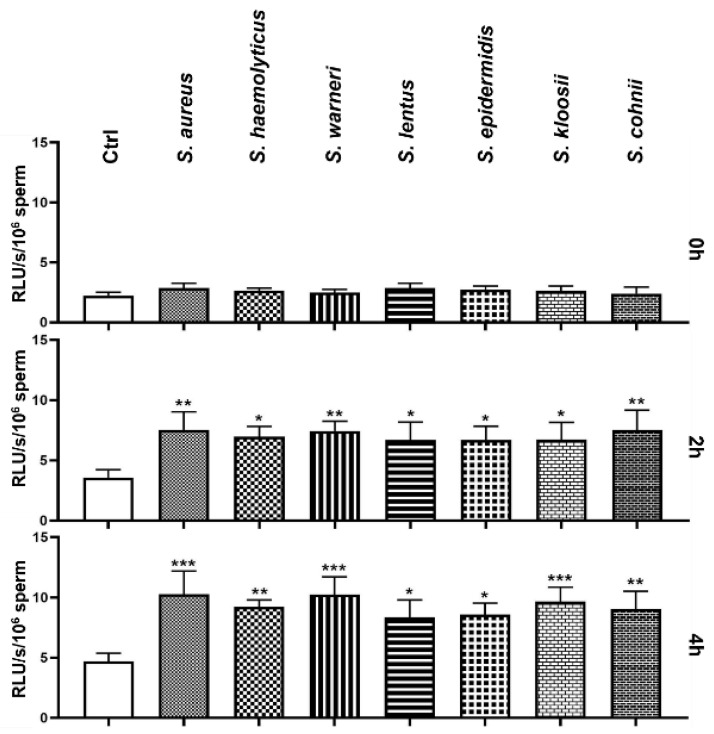
Changes in the global reactive oxygen species production during the 4 h bacteria/sperm co-incubation in vitro at 37 °C. Each group treated with *Staphylococcus* species was compared with the Ctrl group with no added bacteria. Each bar represents the mean value of relative light units per second per 10^6^ sperm cells ± S.E.M. Five individual experiments were performed to obtain these data. The level of statistical significance was set at * *p* < 0.05; ** *p* < 0.01; *** *p* < 0.001.

During the initial measurement of ROS production, none of the experimental groups significantly differed from the Ctrl group. However, significantly increased ROS production was observed after 2 h in every group treated with bacteria (Figure 4). Especially, the groups affected by *S. aureus*, *S. warneri*, and *S. cohnii* significantly exceeded (*p* < 0.01) the Ctrl group in ROS production. Similarly to the 2 h measurement, the groups treated with *S. aureus*, *S. warneri*, and *S. kloosii* exhibited the highest ROS levels amongst the observed groups, which significantly increased (*p* < 0.001) when compared to the Ctrl group. Furthermore, the presence of *S. haemolyticus*, *S. cohnii* (*p* < 0.01), *S. lentus*, and *S. epidermidis* (*p* < 0.05) significantly increased the ROS production.

### 3.5. Effect of Staphylococcus-Induced Bacteriospermia on the DNA Fragmentation in Spermatozoa

**Figure 5:**
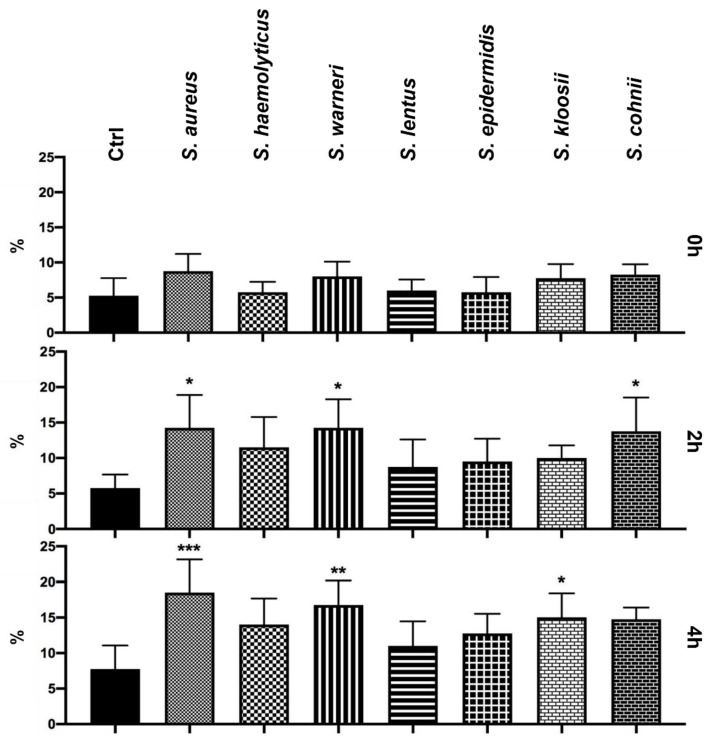
Changes in the sperm DNA fragmentation during the 4 h bacteria/sperm co-incubation in vitro at 37 °C. Each group treated with *Staphylococcus* species was compared with the Ctrl group with no added bacteria. Each bar represents the mean percentage of spermatozoa with the fragmented DNA inside the sperm head ± S.E.M. Five individual experiments were performed to obtain these data. The level of statistical significance was set at * *p* < 0.05; ** *p* < 0.01; *** *p* < 0.001.

Initially, bacteriospermia induced by *Staphylococcus* species did not affect the DNA integrity of spermatozoa. On the other hand, a significantly increased (*p* < 0.05) damage of sperm DNA was recorded after 2 h in the groups infected by *S. aureus*, *S. warneri*, and *S. cohnii* (Figure 5). The final analysis showed a serious disruption of the sperm DNA in case of the groups treated with *S. aureus* (*p* < 0.001), *S. warneri* (*p* < 0.01), and *S. kloosii* (*p* < 0.05). However, a growing trend of detrimental effects of *Staphylococcus* species on the DNA intactness was observed in each experimental group when compared to the Ctrl group.

### 3.6. Effect of Staphylococcus-Induced Bacteriospermia on Extracellular Concentration of Mg

**Figure 6:**
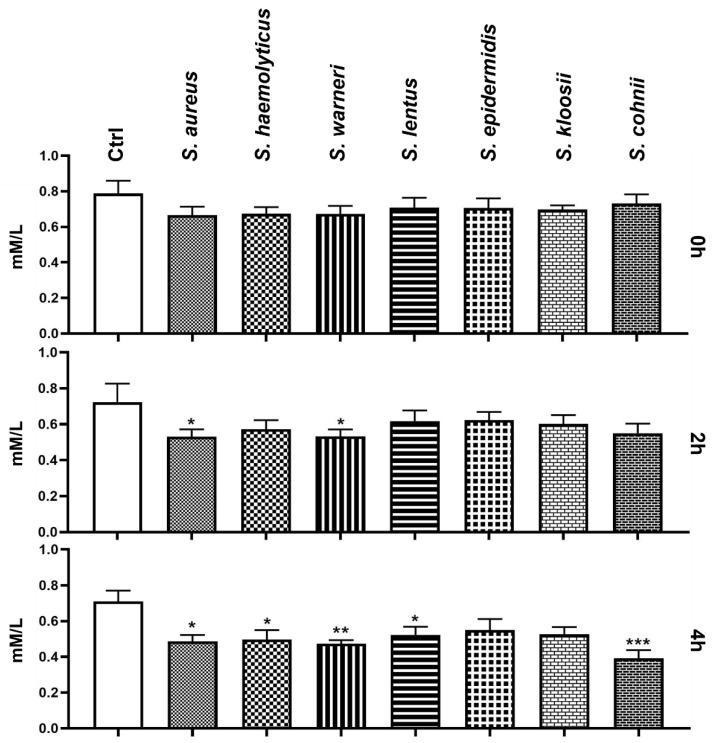
Changes in concentration of extracellular Mg during the 4 h bacteria/sperm co-incubation in vitro at 37 °C. Each group treated with *Staphylococcus* species was compared with the Ctrl group with no added bacteria. Each bar represents the mean concentration of extracellular Mg ± S.E.M. Five individual experiments were performed to obtain these data. The level of statistical significance was set at * *p* < 0.05; ** *p* < 0.01; *** *p* < 0.001.

During the first measurement, Mg content in the experimental groups was comparable with the Mg content in the Ctrl group. In comparison to Ctrl, significantly lower levels of Mg were detected after 2 h of co-incubation with *S. aureus* and *S. warneri* (*p* < 0.05). Subsequent analysis after 4 h showed a notable decrease in Mg concentration in several experimental groups (Figure 6), mainly when treated with *S. cohnii* (*p* < 0.001), *S. warneri* (*p* < 0.01), *S. aureus*, *S. haemolyticus*, and *S. lentus* (*p* < 0.05).

### 3.7. Effect of Staphylococcus-Induced Bacteriospermia on Extracellular Concentration of Ca

**Figure 7:**
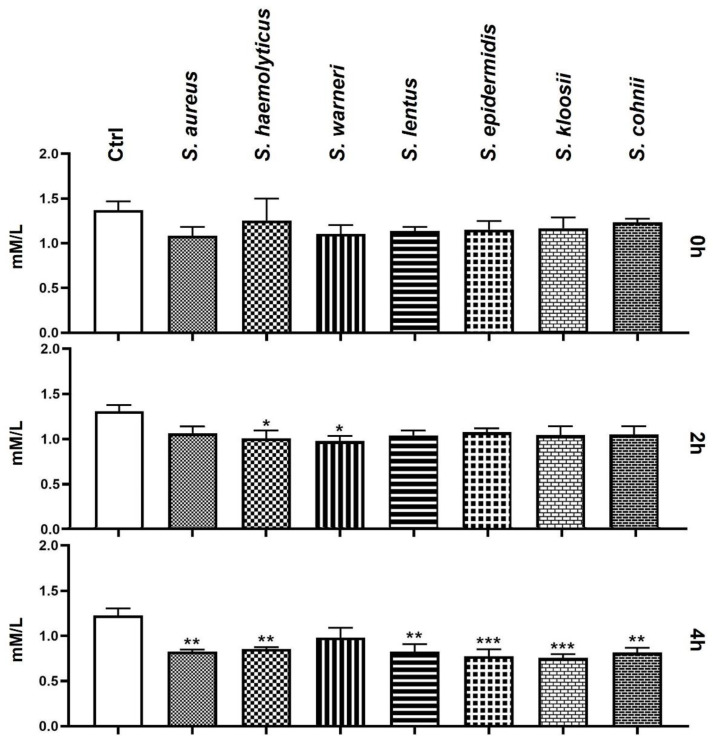
Changes in concentration of extracellular Ca during the 4 h bacteria/sperm co-incubation in vitro at 37 °C. Each group treated with *Staphylococcus* species was compared with the Ctrl group with no added bacteria. Each bar represents the mean concentration of extracellular Ca ± S.E.M. Five individual experiments were performed to obtain these data. The level of statistical significance was set at * *p* < 0.05; ** *p* < 0.01; *** *p* < 0.001.

Similar to the Mg content measurement, the initial analysis of Ca concentration did not show any differences in comparison to the Ctrl group. After 2 h of the bacteria/sperm co-incubation, the groups treated with *S. warneri* and *S. haemolyticus* exhibited significantly lower extracellular Ca concentration compared to Ctrl. After 4 h, almost every experimental group, including the groups containing *S. epidermidis*, *S. kloosii* (*p* < 0.001), *S. aureus*, *S. haemolyticus*, *S. lentus*, and *S. cohnii* (*p* < 0.01), presented with a significant decrease in Ca content when compared to the Ctrl (Figure 7).

## 4. Discussion

This study was built on the premises of our previous studies which reported that *Staphylococcus* species are highly represented in bovine semen with a decline in spermatozoa quality [1,2,11]. Semen samples were processed using the Percoll^®^ Plus density gradient medium and separated sperm pellets were individually contaminated by the *Staphylococcus* species in the presence of DPBS with MgCl_2_ and CaCl_2_. Single-layer centrifugation using colloids was previously carried out to remove bacteria [17] and dead cells [18] from ejaculates. Therefore, our study did not compare bacterial load of neat ejaculate and Percoll-processed sperm nor the representation of dead cells in sperm pellets.

*Staphylococcal*-simulated in vitro infection clearly proved the negative effect on bovine spermatozoa motility. On the other hand, the motility decrease did not occur immediately after the bacteria/sperm contact but it came along with the elapsing time. Similarly, Fraczek et al. [19] assessed the fertilizing potential of human spermatozoa during in vitro semen infection and inflammation. Isolates of *Escherichia coli*, *Staphylococcus haemolyticus*, and *Bacteroides ureolyticus* from bacteriospermic and leukocytospermic patients were co-incubated with spermatozoa originating from healthy volunteers, while impaired sperm quality, including a decreased progressive motility, increased the ratio of necrotic cells. Furthermore, membrane damage as well as lipid peroxidation were observed after 2 h in their study. Furthermore, mutual incubation of bacteria and leukocytes with sperm was reflected in an enhanced negative effect on the sperm quality. In contrast, our study did not record changes in the sperm plasma membrane integrity between bacteria-treated and bacteria-untreated cells when evaluated using the eosin–nigrosin staining protocol. This discrepancy could be explained by the single-layer centrifugation when only the selected-quality sperm were used for our experiments. Another study [20] revealed that ROS generation is probably behind the detrimental effects of bacterial infection on the spermatozoa quality, while leukocytes-mediated oxidative stress is not necessary to invoke significant changes in the phospholipid bilayer of sperm membrane. Our results also confirmed that only the individual effect of bacteriospermia may disrupt oxidative balance in bovine semen. Several other studies also reported a significant decrease in the sperm motility in the presence of *S. aureus* [21,22]. The pathogenicity of *S. aureus* may lie in its property to adhere directly through its cell wall to the host cell without any pili-like structures [23]. Moreover, α-toxin released by *S. aureus* form small pores in the sperm membrane may lead to uncontrolled Na^+^ flux into the cell and the initiation of apoptosis [24].

However, there was another factor diminishing the spermatozoa motility. Prabha et al. [25] isolated the sperm immobilization factor (SIF) from *S. aureus*, which causes immobilization of spermatozoa, and thus provided another possible explanation for the motility decrease during our experiments. Subsequently, it was found that this factor also inhibits mitochondrial Mg^2+^-ATPase activity [26]. Similarly, a sperm agglutinating factor (SAF) was previously isolated from *S. warneri* and its recombinant version was recently proved as spermicidal in vitro, but also as an excellent contraception in vivo [27]. As mitochondrial membrane potential may predict 4-h sperm motility [28], intact mitochondria are an essential factor of sperm motion. Our research showed that semen samples infected mainly by *S. aureus*, *S. warneri*, *S. cohnii*, and *S. kloosii* were distinguished by the reduced mitochondrial membrane potential. A significantly higher expression of early and late apoptotic markers, including lower mitochondrial membrane potential and phosphatidylserine externalization, was recorded in human bacteriospermic semen samples, while *Staphylococcus* species were predominantly represented [10].

Our results showed that all tested bacterial isolates significantly increased the concentration of ROS. The most significant overproduction was measured when semen samples were infected by *S. aureus*, *S. warneri*, and *S. kloosii*. Oxidative insults were directly reflected in the sperm DNA fragmentation analysis, when the highest percentage of spermatozoa with damaged DNA was observed just in these three groups. This suggests that oxidative stress is a direct cause of DNA damage in spermatozoa during *Staphylococcus*-induced bacteriospermia. Moreover, it seemed that every applied isolate would significantly increase ROS levels and subsequent sperm DNA damage at higher bacterial concentrations.

Our previous study [11] found out correlations between the presence of bacteria in bovine ejaculates and biochemical composition of seminal plasma. Especially, Mg seminal concentration acted as a critical parameter in terms of the bacterial presence. Current research revealed a significant descend of extracellular Mg concentration when bovine semen samples were treated with several *Staphylococcus* species, including *S. aureus* and *S. warneri* during the first 2 h, possibly signaling the consumption of this most represented divalent cation in biologic systems by the studied bacterial species. A possible utilization of magnesium from the extracellular matrix support numerous magnesium sensors and transporters with which bacteria are equipped [29]. The role of magnesium in the cell physiology is irreplaceable as an important cofactor of enzymatic reactions, including respiration, glycolysis, proteosynthesis, and reproduction. The presence of magnesium is necessary to maintain the impermeability of mitochondrial membrane [30] and it is the main antagonist of Ca^2+^. Their ratio may affect the semen quality [12]. Another study directly indicates that extracellular Mg^2+^ concentration affected the sperm motility and the process of hyperactivation [31]. Calcium plays a key role in sperm motility, acrosome reaction, and capacitation. A lack of this cation was previously observed in semen from infertile men suffering from asthenozoospermia [32].

This study pointed out which of the *Staphylococcus* species could represent a risk in case of overgrowth. Although such high concentrations are commonly not present in the insemination straws, we suggest to target the antibacterial supplementation of insemination doses against the selected species. Especially, bacterial species resistant to frequently used antibiotics may represent a high risk [33]. For the future, it would be interesting to monitor intracellular Mg and Ca concentration in order to investigate if the environment with a reduced Mg and Ca concentration would be directly reflected in reduced Mg and Ca amounts inside the cell, and thus a lower availability of these cations for the metabolic processes.

## 5. Conclusions

In conclusion, colonization of bovine spermatozoa by *Staphylococcus* species led to detrimental effects on the structural as well as functional components of the sperm cells. Bacteriospermia induced by *Staphylococcus* species contributed to increased ROS levels already after 2 h of co-incubation with every tested species. Oxidative insults resulted in an increased rate of the sperm with fragmented DNA and altered mitochondrial membrane potential, which was particularly observed in the groups infected with *S. aureus*, *S. warneri*, *S. kloosii*, and *S. cohnii*. However, the presence of all tested bacteria significantly decreased the sperm motility. Moreover, our results revealed a significant loss of extracellular magnesium and calcium in the groups treated by the *Staphylococcus* species. This study revealed the species that represent the highest risk to the sperm quality, and therefore the antimicrobial supplementation should focus on these species predominantly. Further studies are needed in order to understand the mechanisms and consequences behind Mg and Ca loss during bacteriospermia.

## Data Availability

The data presented in this study are available on request from the corresponding author.
